# Ultrasound-Assisted Osmotic Pretreatment of Myrtle (*Myrtus communis* L.) Berries with Grape Molasses: Impacts on Convective Drying Quality

**DOI:** 10.3390/plants15172616

**Published:** 2026-08-27

**Authors:** Sevinç Ateş, Ismail Boyar, Esra Gölcü, Doğan Ergün, Luca Mazzoni, Ebru Kafkas, Can Ertekin, Deniz Hazar

**Affiliations:** 1 Department of Horticulture, Faculty of Agriculture, Akdeniz University, Antalya 07058, Türkiye; ssener@akdeniz.edu.tr; 2 Department of Agricultural Machinery and Technologies Engineering, Faculty of Agriculture, Akdeniz University, Antalya 07070, Türkiye; ismailboyar@akdeniz.edu.tr (I.B.); ertekin@akdeniz.edu.tr (C.E.); 3 Department of Horticulture, Faculty of Agriculture, Recep Tayyip Erdoğan University, Rize 53300, Türkiye; esra.golcu@erdogan.edu.tr; 4 Department of Horticulture, Faculty of Agriculture, Çukurova University, Adana 01330, Türkiye; dergun@cu.edu.tr (D.E.); ebru@cu.edu.tr (E.K.); 5 Department of Agricultural, Food, and Environmental Sciences, Università Politecnica Delle Marche, 60121 Ancona, Italy; l.mazzoni@staff.univpm.it

**Keywords:** *Myrtus communis*, ultrasound-assisted osmotic pretreatment, grape molasses, forced-air convective drying, total anthocyanin content, phenolic compounds

## Abstract

Myrtle berries are rich in bioactive phenolics and volatile terpenes and have potential for use in functional food products. However, the combined application of grape molasses and ultrasound as a pretreatment before drying has received limited attention. This study characterized fresh ‘Yakup’ myrtle berries as baseline material and explored the drying behavior and measured quality attributes of berries subjected to twelve pretreatments combining grape molasses or water, ultrasound-assisted or static immersion, and treatment durations of 30, 45, or 60 min. Pretreated and untreated fruits were dried by forced-air convection at 60, 75, and 90 °C. Dried samples were evaluated for antioxidant activity, total phenolic content (TPC), total anthocyanin content (TAC), sugar composition, and drying behavior. The shortest observed drying times at 60 and 75 °C occurred after ultrasound treatment in grape molasses for 30 and 45 min, respectively, whereas static immersion in grape molasses for 60 min and the untreated control showed the shortest drying times at 90 °C. Static immersion in grape molasses for 45 min showed the highest measured TPC at 60 °C (1353.47 mg GAE/100 g dw), while the untreated control showed the highest TPC at 75 and 90 °C and the highest TAC at all three temperatures. The treatment showing the highest total sugar concentration also differed with drying temperature. Thus, no single pretreatment consistently maximized all measured attributes. The findings identify condition-specific candidate strategies rather than a universally superior pretreatment. Because each treatment–temperature combination was represented by one drying tray and the triplicate compositional determinations were analytical rather than independent process replicates, the comparisons are descriptive and require confirmation in independently replicated processing trials. Complete pretreatment mass balances are also required before water loss, solid gain, solute uptake, or true compound retention can be quantified. Looking ahead, studies can be conducted that include sensory and economic evaluations by creating customized pretreatment protocols for industrial applications.

## 1. Introduction

*Myrtus communis* L., commonly known as myrtle, is an emblematic evergreen shrub of the Mediterranean flora, valued since antiquity for its aromatic, medicinal, and culinary properties [1,2]. Beyond these traditional uses, myrtle holds significant ornamental value. Its attractive evergreen foliage, abundant summer flowering, and persistent fruits make it a popular choice for Mediterranean gardens, used as hedges, potted plants, and even for bonsai [3,4]. It is also an ideal cut foliage for bouquets and wreaths with its long-lasting, pleasant fragrance, dark green, small and glossy leaves. Its dark blue-black berries are particularly rich in health-promoting phytochemicals, including diverse polyphenols (e.g., myricetin derivatives, ellagitannins), anthocyanins (mainly delphinidin and petunidin glycosides), vitamins, and essential oils [5,6,7]. These compounds confer potent antioxidant, anti-inflammatory, and antimicrobial activities, positioning myrtle berries as a promising raw material for functional foods and nutraceuticals [6,8,9]. Myrtle berries have also been processed into confectionery and juice products [10,11], supporting interest in the development of new food-processing applications.

However, even with a relatively robust structure compared to very delicate berries, the shelf life of fresh myrtle fruits under ambient conditions is limited. Drying is a common and effective preservation method to extend availability, reduce weight and volume, and help preserve their valuable bioactive compounds for year-round use [12,13]. Conventional hot-air drying, while practical, often involves prolonged exposure to high temperatures, which can lead to detrimental effects on product quality, such as degradation of thermolabile nutrients (e.g., anthocyanins, vitamins), loss of volatile aroma compounds, undesirable color changes (browning), and texture hardening [14,15]. Therefore, developing strategies to shorten drying time while maintaining the quality attributes of the dried product is important for producing high-value dried myrtle products.

Pretreatment techniques before drying have been explored to reduce drying time and limit quality losses [16]. Osmotic pretreatment and its combination with ultrasound are promising approaches for fruit processing [17,18]. Immersion in a hypertonic medium may promote simultaneous water loss and solute gain [19], while ultrasound-induced cavitation can alter tissue structure and facilitate mass transfer [18,20]. Grape molasses was selected in the present study as a food-grade, high-soluble-solids natural medium with regional relevance, rather than as a model sucrose solution. Previous studies using natural molasses-based osmotic media support this processing concept [21,22]. Nevertheless, water loss, solid gain, and the transfer of specific molasses constituents must be measured directly before enrichment or mechanistic claims can be made.

Despite the documented benefits of UAOP, its application to myrtle berries, especially using a natural osmotic agent like grape molasses, remains unexplored. Furthermore, the combined impact of such a novel pretreatment with varying drying air temperatures on the comprehensive quality profile of myrtle berries, including dried-product bioactive-compound contents and sugar composition, has not been systematically investigated. Volatile compounds are important determinants of myrtle aroma and may be affected by postharvest conditions [6,23]. Therefore, the fresh-fruit volatile profile was included solely as baseline characterization for future processing studies; it was not used to infer aroma retention or changes caused by pretreatment and drying.

The present study aimed to investigate the effects of twelve different pretreatments, combining two media (grape molasses and water), two application methods (ultrasound-assisted and static immersion), and three durations (30, 45, and 60 min), followed by forced-air convective drying at three temperatures (60, 75, and 90 °C), on ‘Yakup’ myrtle berries. The specific objectives were to (1) evaluate the effects of these processing conditions on the measured total phenolic content, total anthocyanin content, and DPPH antioxidant activity of dried berries; (2) determine the sugar profiles of the dried berries; (3) characterize the sugar, organic acid, and volatile profiles of fresh berries as baseline information; and (4) compare drying behavior under different pretreatment and temperature combinations. The findings were intended to provide information for selecting processing conditions according to specific dried-product quality attributes.

## 2. Results

### 2.1. Pomological, Physicochemical, and Bioactive Characteristics of Fresh Fruits

The initial characteristics of the fresh ‘Yakup’ myrtle fruits are presented in Table 1. The fruits had an average weight of 0.47 g with dimensions of 8.64 mm in width and 9.02 mm in length. The color values indicated a dark hue (L* = 30.23) with notable red and yellow pigmentation (a* = 6.30, b* = 7.85). The fruits exhibited a high soluble solids content (17.82 Brix), moderate acidity (0.97% malic acid), and a slightly acidic pH (4.23).

The bioactive compound profile revealed high total phenolic content (605.99 mg GAE/100 g fw) and considerable antioxidant activity (86.63% DPPH inhibition, equivalent to 77.03 μmol TE/100 g fw). The total anthocyanin content averaged 94.56 mg cyanidin-3-glucoside equivalent per liter of fresh juice. These measurements provide baseline characterization of fresh ‘Yakup’ myrtle berries. Because fruits collected from the three trees were pooled before allocation to the drying treatments, tree-specific variation was not retained as independent biological replication in the drying experiment.

### 2.2. Sugar, Organic Acid, and Volatile Profile

The sugar, organic acid, and volatile compound composition of fresh fruits is summarized in Table 2. Glucose and fructose were the predominant sugars (5.15 and 5.70 g/100 mL, respectively), with a fructose-to-glucose ratio of ~1.11, contributing to the balanced sweet taste. Sucrose was not detected in fresh fruits. Succinic acid was the predominant organic acid (2.63 g/100 mL, ~65% of total), followed by malic and citric acids—a distinctive feature of the ‘Yakup’ cultivar.

The volatile aroma profile was dominated by oxygenated monoterpenes, particularly eucalyptol (41.14%) and α-terpineol (5.55%), with total alcohols representing ~60% of volatiles. α-Pinene (12.75%) was the major terpene hydrocarbon. Esters contributed 13.12%, primarily (-)-8-p-menthen-2-yl acetate and linalyl acetate. This camphoraceous-minty profile provides baseline characterization of the fresh fruit. Volatile changes following pretreatment and drying were not evaluated in the present study.

### 2.3. Bioactive-Compound Profiles Among Pretreatment–Temperature Combinations

The measured DPPH antioxidant activity, total phenolic content, and total anthocyanin content varied among the pretreatment–temperature combinations. Because each combination was represented by one dried sample and the triplicate measurements were analytical determinations of that sample, the results are presented descriptively in Table 3, Table 4 and Table 5. No inferential comparisons were performed.

#### 2.3.1. Antioxidant Activity (DPPH)

DPPH radical-scavenging activity ranged from 78.22% to 83.93% inhibition across the pretreatment–temperature combinations, corresponding to 69.93–75.64 µmol TE/100 g dw (Table 3). At 60 °C, the highest measured inhibition values were observed in T2 (81.12%), T6 (81.10%), and T11 (81.04%). At 75 °C, T3 showed the highest measured value (83.93%), whereas T2 showed the highest value at 90 °C (81.88%). Thus, no single pretreatment consistently produced the highest measured DPPH activity at all three drying temperatures.

The fresh-fruit DPPH value of 86.63% is presented separately as baseline characterization. Because the fresh and dried samples were expressed on different analytical bases, antioxidant-activity retention was not calculated. The differences among the dried samples describe the analyzed pretreatment–temperature combinations and should not be interpreted as statistically demonstrated treatment effects.

**Table 3:** Antioxidant activity (DPPH) of dried myrtle fruits.

Pretreatment *	60 °C	75 °C	90 °C
% Inhibition/µmol TE/100 g dw *			
T1 (GM + US 30)	79.29 ± 0.49/71.00 ± 0.49	80.97 ± 0.28/72.69 ± 0.28	80.88 ± 0.63/72.60 ± 0.63
T2 (GM + US 45)	81.12 ± 0.66/72.84 ± 0.66	81.80 ± 0.54/73.51 ± 0.54	81.88 ± 0.94/73.59 ± 0.94
T3 (GM + US 60)	78.22 ± 0.62/69.93 ± 0.62	83.93 ± 2.73/75.64 ± 2.73	80.72 ± 0.15/72.38 ± 0.17
T4 (W + US 30)	78.93 ± 0.02/70.64 ± 0.02	80.26 ± 0.69/71.98 ± 0.69	79.28 ± 0.48/70.99 ± 0.48
T5 (W + US 45)	78.90 ± 0.78/70.61 ± 0.78	81.24 ± 0.18/72.96 ± 0.18	80.46 ± 0.18/72.17 ± 0.18
T6 (W + US 60)	81.10 ± 0.85/72.82 ± 0.85	80.41 ± 0.82/72.13 ± 0.82	78.53 ± 0.06/70.24 ± 0.06
T7 (GM + N 30)	80.82 ± 0.27/72.53 ± 0.27	81.21 ± 0.51/72.92 ± 0.51	79.07 ± 0.98/70.79 ± 0.98
T8 (GM + N 45)	79.06 ± 0.66/70.77 ± 0.66	81.64 ± 0.07/73.35 ± 0.07	80.13 ± 0.78/71.77 ± 0.85
T9 (GM + N 60)	79.15 ± 0.48/70.86 ± 0.48	81.24 ± 0.08/72.96 ± 0.08	78.65 ± 0.61/70.36 ± 0.61
T10 (W + N 30)	79.84 ± 0.40/71.55 ± 0.40	80.10 ± 1.13/71.81 ± 1.13	79.37 ± 0.52/71.08 ± 0.52
T11 (W + N 45)	81.04 ± 0.51/72.75 ± 0.51	79.38 ± 0.45/71.09 ± 0.45	80.34 ± 0.42/72.05 ± 0.42
T12 (W + N 60)	80.25 ± 0.61/71.97 ± 0.61	80.78 ± 1.13/72.49 ± 1.13	80.70 ± 0.02/72.41 ± 0.02
T13 (Control)	80.41 ± 2.06/72.13 ± 2.06	81.23 ± 0.98/72.95 ± 0.98	80.49 ± 0.09/72.21 ± 0.09

* Values are expressed as mean ± SD of three analytical determinations performed on the same dried sample for each pretreatment × drying-temperature combination. These measurements represent analytical replicates rather than independent experimental replicates; therefore, no inferential statistical comparisons were performed. GM: grape molasses; W: water; US: ultrasound-assisted; N: static immersion; dw: dry weight.

#### 2.3.2. Total Phenolic Content (TPC)

The measured TPC concentrations ranged from 808.10 to 1415.27 mg GAE/100 g dw across the pretreatment–temperature combinations (Table 4). At 60 °C, T8 showed the highest measured TPC concentration (1353.47 mg GAE/100 g dw), followed by T4 (1315.15 mg GAE/100 g dw) and the untreated control, T13 (1247.77 mg GAE/100 g dw). At 75 °C, the highest value was observed in T13 (1415.27 mg GAE/100 g dw), followed by T2 (1313.40 mg GAE/100 g dw). At 90 °C, T13 again showed the highest measured concentration (1403.83 mg GAE/100 g dw), followed by T6 (1389.10 mg GAE/100 g dw) and T12 (1318.85 mg GAE/100 g dw).

The temperature-related pattern varied among pretreatments. For example, T6 and T12 showed their highest measured concentrations at 90 °C, whereas T4 and T8 showed their highest values at 60 °C. These results represent extractable phenolic concentrations per unit dry weight and should not be interpreted as statistically demonstrated treatment effects or as improved phenolic preservation relative to fresh fruit.

**Table 4:** Total Phenolic Content (TPC) of Dried Myrtle Fruits (mg GAE/100 g dw).

Pretreatment *	60 °C	75 °C	90 °C
T1 (GM + US 30)	1152.02 ± 4.34	1084.66 ± 24.00	1137.21 ± 8.38
T2 (GM + US 45)	1040.74 ± 7.16	1313.40 ± 6.36	1276.16 ± 22.26
T3 (GM + US 60)	925.18 ± 7.70	966.56 ± 1.91	881.69 ± 1.17
T4 (W + US 30)	1315.15 ± 2.23	949.60 ± 6.79	1252.95 ± 22.20
T5 (W + US 45)	938.97 ± 12.51	1006.76 ± 2.82	1009.54 ± 4.62
T6 (W + US 60)	1081.87 ± 12.92	999.37 ± 1.21	1389.10 ± 0.49
T7 (GM + N 30)	1156.23 ± 7.20	1115.08 ± 18.85	1278.77 ± 33.66
T8 (GM + N 45)	1353.47 ± 1.60	1127.23 ± 1.52	1226.46 ± 6.97
T9 (GM + N 60)	896.27 ± 13.94	1102.89 ± 4.20	1146.25 ± 1.41
T10 (W + N 30)	1209.12 ± 0.35	1058.04 ± 11.64	1168.54 ± 19.30
T11 (W + N 45)	808.10 ± 5.94	874.98 ± 7.73	954.21 ± 14.48
T12 (W + N 60)	848.47 ± 11.37	1053.83 ± 10.14	1318.85 ± 25.48
T13 (Control)	1247.77 ± 4.47	1415.27 ± 9.93	1403.83 ± 23.08

* Values are expressed as mean ± SD of three analytical determinations performed on the same dried sample for each pretreatment × drying-temperature combination. These measurements represent analytical replicates rather than independent experimental replicates; therefore, no inferential statistical comparisons were performed. GM: grape molasses; W: water; US: ultrasound-assisted; N: static immersion; dw: dry weight.

#### 2.3.3. Total Anthocyanin Content (TAC)

The measured TAC concentrations ranged from 0.80 to 8.53 mg cyanidin-3-glucoside equivalent/100 g dw across the pretreatment–temperature combinations (Table 5). The untreated control (T13) showed the highest measured TAC concentration at each drying temperature, with values of 8.53, 2.50, and 4.36 mg/100 g dw at 60, 75, and 90 °C, respectively. Among the pretreated samples, the highest measured values at 60 °C were observed in T2 (5.48 mg/100 g dw) and T8 (5.39 mg/100 g dw). At 75 °C, T1 and T12 showed the highest measured values among the pretreated samples, whereas at 90 °C the highest values were observed in T12 and T6.

For many pretreatments, the TAC concentration measured at 60 °C was higher than the corresponding values at 75 and 90 °C; however, this pattern was not uniform across all treatments. Because fresh-fruit TAC was expressed per unit volume of juice and dried-sample TAC per unit dry weight, true anthocyanin retention or percentage loss could not be calculated. The observed differences are therefore interpreted descriptively.

**Table 5:** Total anthocyanin content (TAC) of dried myrtle fruits (mg cyanidin-3-glucoside equivalent/100 g dw).

Pretreatment *	60 °C	75 °C	90 °C
T1 (GM + US 30)	4.95 ± 0.11	1.95 ± 0.03	1.16 ± 0.04
T2 (GM + US 45)	5.48 ± 0.09	1.47 ± 0.00	1.46 ± 0.08
T3 (GM + US 60)	2.67 ± 0.04	1.16 ± 0.01	0.80 ± 0.03
T4 (W + US 30)	2.71 ± 0.01	1.66 ± 0.02	2.12 ± 0.01
T5 (W + US 45)	2.07 ± 0.01	1.53 ± 0.09	1.26 ± 0.01
T6 (W + US 60)	2.01 ± 0.08	1.49 ± 0.09	2.21 ± 0.06
T7 (GM + N 30)	4.40 ± 0.07	1.61 ± 0.01	1.72 ± 0.09
T8 (GM + N 45)	5.39 ± 0.07	1.57 ± 0.03	1.78 ± 0.01
T9 (GM + N 60)	3.97 ± 0.08	1.15 ± 0.05	1.61 ± 0.04
T10 (W + N 30)	2.74 ± 0.12	1.72 ± 0.03	1.82 ± 0.06
T11 (W + N 45)	1.27 ± 0.03	1.29 ± 0.02	1.41 ± 0.01
T12 (W + N 60)	2.26 ± 0.08	1.87 ± 0.08	2.26 ± 0.02
T13 (Control)	8.53 ± 0.17	2.50 ± 0.05	4.36 ± 0.03

* Values are expressed as mean ± SD of three analytical determinations performed on the same dried sample for each pretreatment × drying-temperature combination. These measurements represent analytical replicates rather than independent experimental replicates; therefore, no inferential statistical comparisons were performed. GM: grape molasses; W: water; US: ultrasound-assisted; N: static immersion; dw: dry weight.

### 2.4. Sugar Profiles Among Pretreatment–Temperature Combinations

The measured sucrose, glucose, fructose, and total sugar concentrations varied among the 39 pretreatment–temperature combinations. Because the replicate measurements were analytical determinations performed on the same dried sample, the sugar results are presented descriptively in Table 6, Table 7, Table 8 and Table 9 without inferential statistical comparisons.

#### 2.4.1. Sucrose Content

Sucrose was not detected in fresh-fruit juice but was quantified in all dried samples, including the untreated control (T13) (Table 6). At 60 °C, T13 showed the highest measured sucrose concentration (1464.50 mg/100 g dw), followed by T7 (1321.81 mg/100 g dw). At 75 °C, the highest measured value was observed in T6 (1154.76 mg/100 g dw), whereas T8 showed the highest concentration at 90 °C (1268.67 mg/100 g dw). T3 showed measured sucrose concentrations of 1048.53, 932.42, and 664.63 mg/100 g dw at 60, 75, and 90 °C, respectively.

The temperature-related response varied among pretreatments. For example, T1 increased from 474.67 mg/100 g dw at 60 °C to 1059.86 mg/100 g dw at 75 °C, whereas T8 showed its lowest measured value at 75 °C and its highest value at 90 °C. Because the untreated control contained sucrose at all three temperatures and showed the highest value at 60 °C, the occurrence of sucrose in the dried samples cannot by itself be interpreted as evidence of uptake from grape molasses. These data describe measured sucrose concentrations and do not establish retention, enrichment, uptake, or thermal degradation.

**Table 6:** Sucrose content of dried myrtle fruits (mg/100 g dw).

Pretreatment *	60 °C	75 °C	90 °C
T1 (GM + US 30)	474.67 ± 17.10	1059.86 ± 61.85	745.22 ± 16.67
T2 (GM + US 45)	968.05 ± 18.13	975.17 ± 42.52	741.94 ± 12.00
T3 (GM + US 60)	1048.53 ± 5.90	932.42 ± 49.68	664.63 ± 21.52
T4 (W + US 30)	873.42 ± 64.76	824.06 ± 22.92	1027.58 ± 43.00
T5 (W + US 45)	1057.46 ± 59.89	970.39 ± 11.18	1039.15 ± 19.57
T6 (W + US 60)	1043.16 ± 54.45	1154.76 ± 35.73	1121.94 ± 7.16
T7 (GM + N 30)	1321.81 ± 56.53	1017.75 ± 86.21	786.05 ± 370.43
T8 (GM + N 45)	1017.12 ± 35.05	681.21 ± 36.59	1268.67 ± 12.15
T9 (GM + N 60)	889.82 ± 3.78	729.68 ± 10.40	1085.74 ± 16.29
T10 (W + N 30)	882.91 ± 0.48	1025.25 ± 180.54	1061.69 ± 13.94
T11 (W + N 45)	844.65 ± 40.19	922.61 ± 38.60	1045.22 ± 13.56
T12 (W + N 60)	815.51 ± 10.59	712.13 ± 24.74	1098.47 ± 28.22
T13 (Control)	1464.50 ± 23.82	863.87 ± 14.68	1095.58 ± 5.31

* Values are expressed as mean ± SD of three analytical determinations performed on the same dried sample for each pretreatment × drying-temperature combination. These measurements represent analytical replicates rather than independent experimental replicates; therefore, no inferential statistical comparisons were performed. GM: grape molasses; W: water; US: ultrasound-assisted; N: static immersion; dw: dry weight.

#### 2.4.2. Glucose Content

Glucose concentrations ranged from 12,901.81 to 23,407.30 mg/100 g dw across the pretreatment–temperature combinations (Table 7). At 60 °C, T5 showed the highest measured glucose concentration (21,768.96 mg/100 g dw), followed by T7, T4, and T3. At 75 °C, the highest value was observed in T10 (23,407.30 mg/100 g dw), followed by T1, T2, and T3. At 90 °C, the untreated control (T13) showed the highest measured value (20,497.92 mg/100 g dw), followed by T2.

The temperature-related pattern differed among pretreatments. For example, T10 showed its highest measured glucose concentration at 75 °C, whereas T8, T9, T11, and T12 showed their lowest values at that temperature. These results describe glucose concentrations on a dry-weight basis and should not be interpreted as statistically demonstrated treatment effects, glucose retention, or confirmed thermal degradation.

**Table 7:** Glucose content of dried myrtle fruits (mg/100 g dw).

Pretreatment *	60 °C	75 °C	90 °C
T1 (GM + US 30)	20,573.98 ± 221.53	22,440.75 ± 462.62	15,532.94 ± 213.19
T2 (GM + US 45)	19,579.50 ± 298.48	22,174.95 ± 594.74	19,979.04 ± 360.65
T3 (GM + US 60)	20,713.06 ± 278.78	22,060.48 ± 500.81	16,986.75 ± 184.34
T4 (W + US 30)	20,942.07 ± 51.22	21,690.86 ± 208.83	17,254.51 ± 62.40
T5 (W + US 45)	21,768.96 ± 255.97	20,542.41 ± 265.99	16,280.69 ± 148.27
T6 (W + US 60)	18,089.94 ± 159.19	21,454.80 ± 16.71	17,551.67 ± 436.72
T7 (GM + N 30)	20,961.93 ± 613.31	20,779.60 ± 126.74	17,965.98 ± 296.20
T8 (GM + N 45)	18,140.10 ± 357.11	15,060.03 ± 47.46	19,627.53 ± 283.70
T9 (GM + N 60)	19,145.99 ± 70.52	14,788.99 ± 133.19	18,024.14 ± 389.87
T10 (W + N 30)	20,117.98 ± 100.61	23,407.30 ± 26.39	17,691.36 ± 314.41
T11 (W + N 45)	20,009.60 ± 36.49	15,870.33 ± 89.74	17,273.89 ± 418.32
T12 (W + N 60)	18,852.11 ± 21.36	12,901.81 ± 109.85	19,419.03 ± 121.38
T13 (Control)	16,714.00 ± 278.87	20,276.11 ± 206.98	20,497.92 ± 391.14

* Values are expressed as mean ± SD of three analytical determinations performed on the same dried sample for each pretreatment × drying-temperature combination. These measurements represent analytical replicates rather than independent experimental replicates; therefore, no inferential statistical comparisons were performed. GM: grape molasses; W: water; US: ultrasound-assisted; N: static immersion; dw: dry weight.

#### 2.4.3. Fructose Content

Fructose concentrations ranged from 12,971.19 to 22,290.35 mg/100 g dw across the pretreatment–temperature combinations (Table 8). At 60 °C, T4 showed the highest measured fructose concentration (19,578.95 mg/100 g dw). At 75 °C, the highest values were observed in T10 (19,428.76 mg/100 g dw) and T6 (19,391.46 mg/100 g dw). At 90 °C, T2 showed the highest measured concentration (22,290.35 mg/100 g dw), followed by T12, T8, and the untreated control.

Higher fructose concentrations were observed at 90 °C than at 60 °C for several treatments, including T2, T6, T8, T9, T12, and T13; however, the magnitude and direction of the temperature-related response varied among pretreatments. Because the fresh- and dried-sample results were expressed on different analytical bases, these patterns should not be interpreted as fructose retention, accumulation relative to fresh fruit, or confirmed conversion of other sugars.

**Table 8:** Fructose content of dried myrtle fruits (mg/100 g dw).

Pretreatment *	60 °C	75 °C	90 °C
T1 (GM + US 30)	15,541.42 ± 307.46	17,767.44 ± 495.38	17,701.83 ± 364.44
T2 (GM + US 45)	14,837.40 ± 136.07	17,935.53 ± 677.18	22,290.35 ± 403.75
T3 (GM + US 60)	15,516.06 ± 206.57	18,184.65 ± 362.34	19,276.16 ± 79.80
T4 (W + US 30)	19,578.95 ± 175.97	17,908.70 ± 135.41	19,488.03 ± 55.31
T5 (W + US 45)	17,009.12 ± 290.96	18,306.91 ± 244.50	18,484.94 ± 122.30
T6 (W + US 60)	13,554.93 ± 81.03	19,391.46 ± 74.20	19,999.45 ± 331.89
T7 (GM + N 30)	15,979.07 ± 303.08	18,239.73 ± 57.27	19,788.70 ± 119.79
T8 (GM + N 45)	14,016.98 ± 143.13	16,789.29 ± 205.17	21,427.81 ± 103.53
T9 (GM + N 60)	14,802.59 ± 293.92	16,913.83 ± 35.07	20,287.22 ± 433.00
T10 (W + N 30)	15,215.76 ± 82.64	19,428.76 ± 468.82	19,677.23 ± 145.63
T11 (W + N 45)	15,464.17 ± 202.24	18,725.55 ± 95.88	18,232.23 ± 316.39
T12 (W + N 60)	14,748.20 ± 323.62	15,011.94 ± 73.68	21,794.97 ± 506.32
T13 (Control)	12,971.19 ± 122.54	17,595.97 ± 142.58	21,244.74 ± 261.72

* Values are expressed as mean ± SD of three analytical determinations performed on the same dried sample for each pretreatment × drying-temperature combination. These measurements represent analytical replicates rather than independent experimental replicates; therefore, no inferential statistical comparisons were performed. GM: grape molasses; W: water; US: ultrasound-assisted; N: static immersion; dw: dry weight.

#### 2.4.4. Total Sugar Content

Total sugar concentrations ranged from 28.63 to 43.86 g/100 g dw across the pretreatment–temperature combinations (Table 9). The highest measured total sugar concentration at 60 °C was observed in T4 (41.39 g/100 g dw). At 75 °C, T10 showed the highest value (43.86 g/100 g dw), whereas T2 showed the highest measured concentration at 90 °C (43.01 g/100 g dw). The untreated control contained 31.15, 38.74, and 42.84 g/100 g dw total sugar at 60, 75, and 90 °C, respectively. Thus, no single pretreatment produced the highest measured total sugar concentration at all three temperatures.

Fresh-fruit sugars were expressed as mg/100 mL juice, whereas dried-sample sugars were expressed as g/100 g dw. Because these analytical bases are not directly comparable, a fresh-to-dried concentration factor could not be calculated. Furthermore, water loss, solid gain, and treatment-specific mass balances were not quantified during pretreatment; therefore, the contribution of solute uptake from the immersion media could not be determined. The results describe total sugar concentrations in the dried products rather than sugar retention or enrichment relative to fresh fruit.

**Table 9:** Total sugar content of dried myrtle fruits (g/100 g dw).

Pretreatment *	60 °C	75 °C	90 °C
T1 (GM + US 30)	36.59 ± 0.44	41.27 ± 0.87	33.98 ± 0.17
T2 (GM + US 45)	35.38 ± 0.32	41.09 ± 1.31	43.01 ± 0.76
T3 (GM + US 60)	37.28 ± 0.08	41.18 ± 0.77	36.93 ± 0.19
T4 (W + US 30)	41.39 ± 0.19	40.42 ± 0.20	37.77 ± 0.06
T5 (W + US 45)	39.84 ± 0.49	39.82 ± 0.50	35.80 ± 0.28
T6 (W + US 60)	32.69 ± 0.29	42.00 ± 0.04	38.67 ± 0.75
T7 (GM + N 30)	38.26 ± 0.97	40.04 ± 0.08	38.54 ± 0.22
T8 (GM + N 45)	33.17 ± 0.26	32.53 ± 0.20	42.32 ± 0.21
T9 (GM + N 60)	34.84 ± 0.22	32.43 ± 0.15	39.40 ± 0.81
T10 (W + N 30)	36.22 ± 0.08	43.86 ± 0.31	38.43 ± 0.24
T11 (W + N 45)	36.32 ± 0.19	35.52 ± 0.07	36.55 ± 0.09
T12 (W + N 60)	34.42 ± 0.35	28.63 ± 0.11	42.31 ± 0.42
T13 (Control)	31.15 ± 0.34	38.74 ± 0.35	42.84 ± 0.62

* Values are expressed as mean ± SD of three analytical determinations performed on the same dried sample for each pretreatment × drying-temperature combination. These measurements represent analytical replicates rather than independent experimental replicates; therefore, no inferential statistical comparisons were performed. GM: grape molasses; W: water; US: ultrasound-assisted; N: static immersion; dw: dry weight.

### 2.5. Individual Phenolic Compound Profiles of Dried Myrtle Fruits

**Figure 1:**
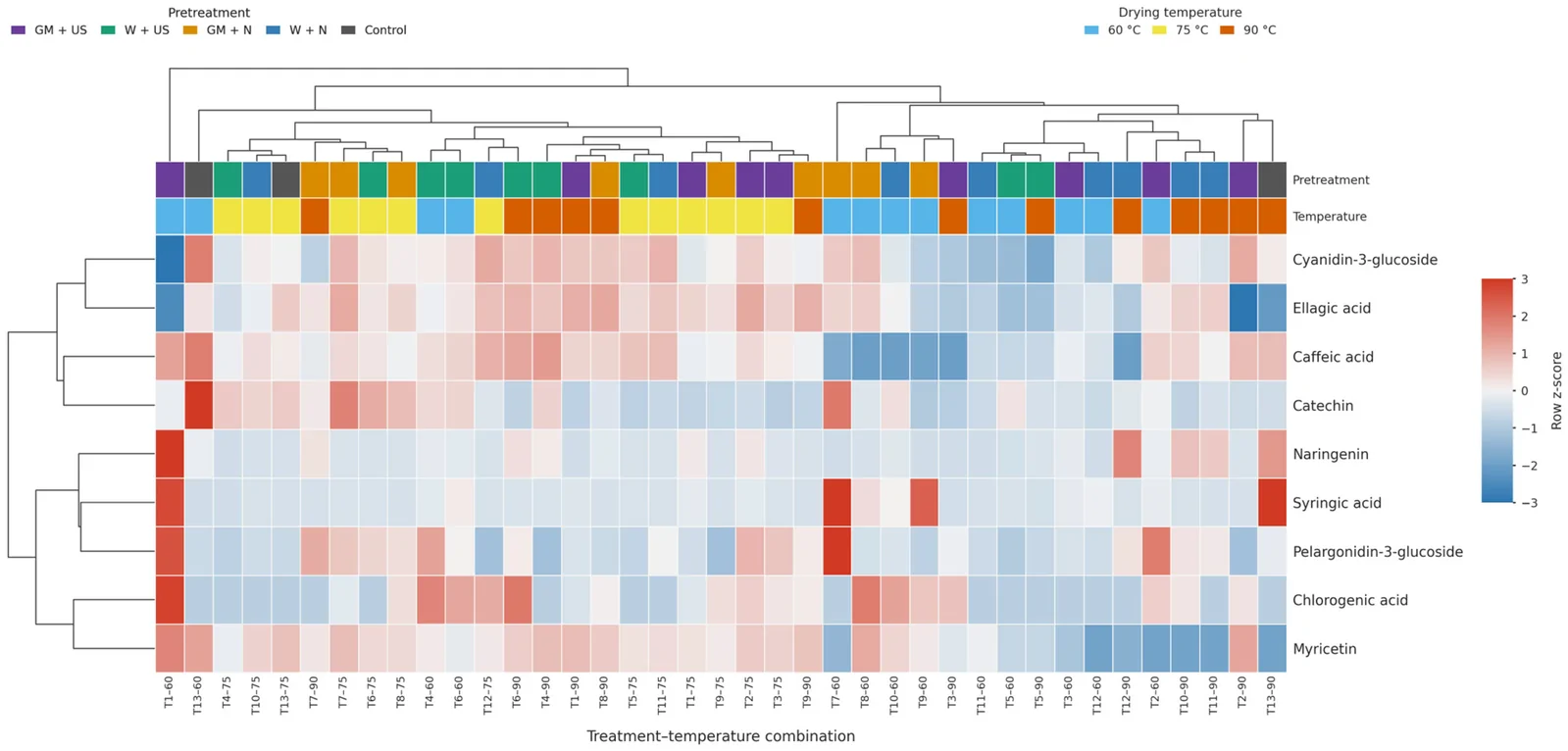
Clustered heatmap of nine individual phenolic compounds in dried myrtle fruits subjected to different pretreatment and drying-temperature combinations. Cell colors represent min–max normalized values calculated separately for each compound, with blue and red corresponding to the minimum and maximum measured concentrations of that compound, respectively. Because normalization was performed separately for each compound, colors indicate relative variation within a compound and should not be used for absolute comparisons among different compounds. The corresponding unscaled concentrations (mg/100 g dw) are provided in Supplementary Table S2. Non-detected observations are reported as n.d. in Supplementary Table S2 and were assigned a value of zero solely for the purpose of heatmap normalization. Each treatment–temperature combination was analyzed once (n = 1); therefore, the heatmap is exploratory and does not support statistical inference, mechanistic interpretation, or conclusions regarding treatment superiority. Dendrograms show hierarchical clustering using Ward’s linkage method and Euclidean distance for compounds (rows) and treatment–temperature combinations (columns).

The concentrations of nine individual phenolic compounds in dried myrtle fruits were visualized using a clustered heatmap after compound-wise min–max normalization (Figure 1). The corresponding unscaled concentration values are provided in Supplementary Table S2. Values recorded as zero in the source analytical dataset represented non-detected compounds and are therefore reported as n.d. in Supplementary Table S2. For heatmap normalization only, these non-detected observations were assigned a numerical value of zero. Because each pretreatment–temperature combination was analyzed once (n = 1), the heatmap is presented solely as an exploratory, hypothesis-generating visualization. Accordingly, no statistical inference, treatment-superiority conclusion, or mechanistic interpretation was derived from these data.

The compound dendrogram assigned the two anthocyanins to different branches. Cyanidin-3-glucoside clustered more closely with ellagic acid, caffeic acid, and myricetin, whereas pelargonidin-3-glucoside occurred in a separate branch together with catechin, naringenin, and syringic acid. Pelargonidin-3-glucoside showed relatively high single-measurement values in several samples dried at 60 °C. In contrast, cyanidin-3-glucoside varied considerably among the pretreatment–temperature combinations and did not exhibit a consistent or monotonic temperature-related pattern. Although its highest individual value was measured in the untreated control at 60 °C (T13; 177.06 mg/100 g dw), similarly high values were observed in several samples dried at 75 and 90 °C. Therefore, no general temperature preference can be inferred from the individual anthocyanin data.

Naringenin showed its highest single-measurement value in T1 at 60 °C (184.82 mg/100 g dw) and relatively high values in T10–T13 at 90 °C, ranging from 37.49 to 72.65 mg/100 g dw. Syringic acid showed elevated values in T1, T7, and T9 at 60 °C and in T13 at 90 °C. Catechin was highly variable, with its highest measured value occurring in T13 at 60 °C (96.31 mg/100 g dw). Ellagic acid, chlorogenic acid, caffeic acid, and myricetin also varied among the pretreatment–temperature combinations. Because these measurements were unreplicated, these observations describe only the analyzed samples and cannot establish treatment effects, thermal stability, compound release, degradation, or other mechanisms.

Hierarchical clustering of the pretreatment–temperature combinations did not produce exclusive groups based solely on drying temperature, immersion medium, application technique, or treatment duration. Instead, combinations representing different temperatures and pretreatment groups were intermingled throughout the column dendrogram. These clusters are therefore descriptive only and should not be interpreted as evidence of treatment similarity or superiority.

Overall, the individual phenolic-compound profiles may be used to generate hypotheses for future research. No validation of the total phenolic or total anthocyanin measurements, practical recommendation, or conclusion regarding treatment superiority was derived from this unreplicated dataset. Future studies should include independent experimental replicates to permit statistical evaluation of individual phenolic-compound responses.

### 2.6. Drying Behavior Under Different Pretreatment–Temperature Conditions

**Figure 2:**
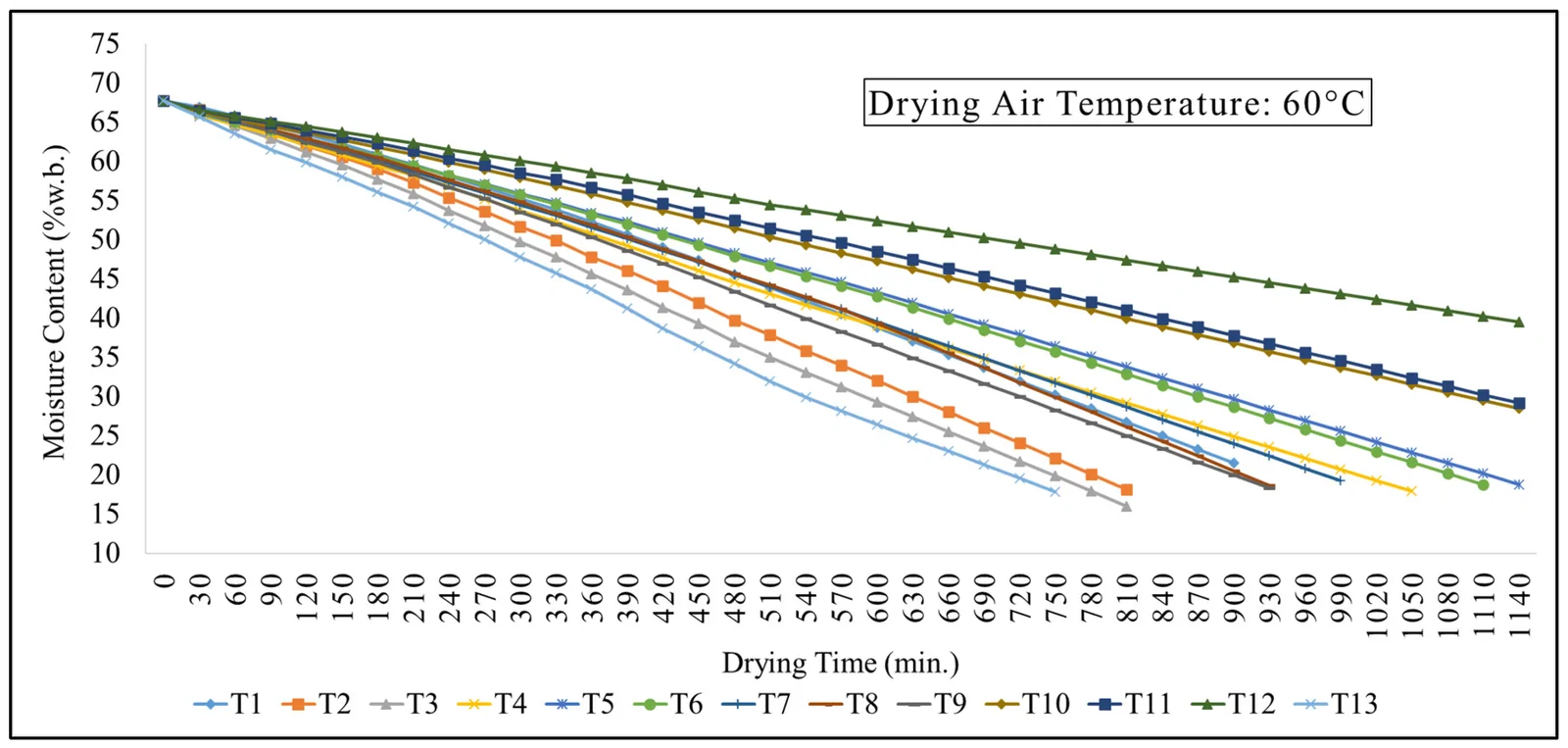
Descriptive drying curves of pretreated and untreated myrtle berries at 60 °C. Each curve represents one labeled tray containing an initial fruit load of 250 g, and mass measurements were recorded at 15 min intervals. All 13 treatment groups were dried simultaneously in the same temperature run. Tray positions were assigned and rearranged using a random-number table. The horizontal reference line indicates the target moisture content of 20% wet basis (wb). The curves represent single drying trays and do not constitute independent process replicates.

The drying curves obtained at 60 °C showed marked differences in moisture removal among the treatments (Figure 2). The initial moisture content was approximately 68% on a wet basis (wb) and decreased progressively during drying. T1 (grape molasses + ultrasound, 30 min) exhibited the fastest drying behavior at this temperature, reaching a moisture content below 20% wb after approximately 780 min. T2 (grape molasses + ultrasound, 45 min), T3 (grape molasses + ultrasound, 60 min), and T4 (water + ultrasound, 30 min) also showed relatively rapid moisture removal, reaching low moisture levels after approximately 810–900 min. In contrast, T12 (water + static immersion, 60 min) exhibited the slowest drying behavior and retained a moisture content of approximately 40% wb after 1140 min. T11 (water + static immersion, 45 min) and T6 (water + ultrasound, 60 min) also showed comparatively slow moisture removal at 60 °C.

**Figure 3:**
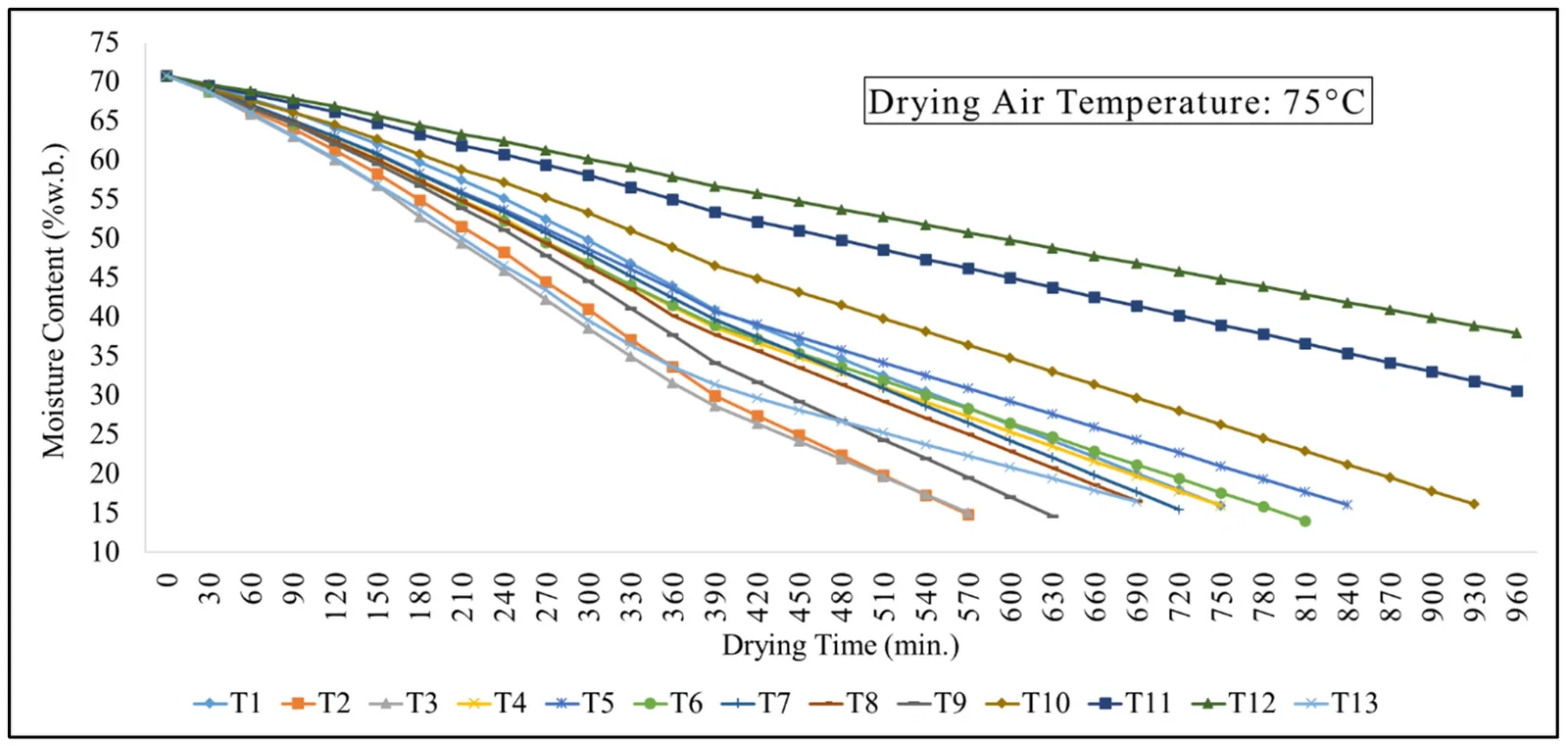
Descriptive drying curves of pretreated and untreated myrtle berries at 75 °C. Each curve represents one labeled tray containing an initial fruit load of 250 g, and mass measurements were recorded at 15 min intervals. All 13 treatment groups were dried simultaneously in the same temperature run. Tray positions were assigned and rearranged using a random-number table. The horizontal reference line indicates the target moisture content of 20% wet basis (wb). The curves represent single drying trays and do not constitute independent process replicates.

At 75 °C, moisture removal was generally faster than at 60 °C (Figure 3). The initial moisture content was approximately 70% wb across the treatments. T2 (grape molasses + ultrasound, 45 min) exhibited the fastest drying behavior, reaching approximately 15–20% moisture content after about 570 min. T1 (grape molasses + ultrasound, 30 min) and T3 (grape molasses + ultrasound, 60 min) reached similarly low moisture levels after approximately 600–630 min. Conversely, T12 (water + static immersion, 60 min) remained at approximately 38% moisture content after 960 min and did not reach the target moisture range during the recorded drying period. T11 (water + static immersion, 45 min) also exhibited comparatively slow moisture removal.

**Figure 4:**
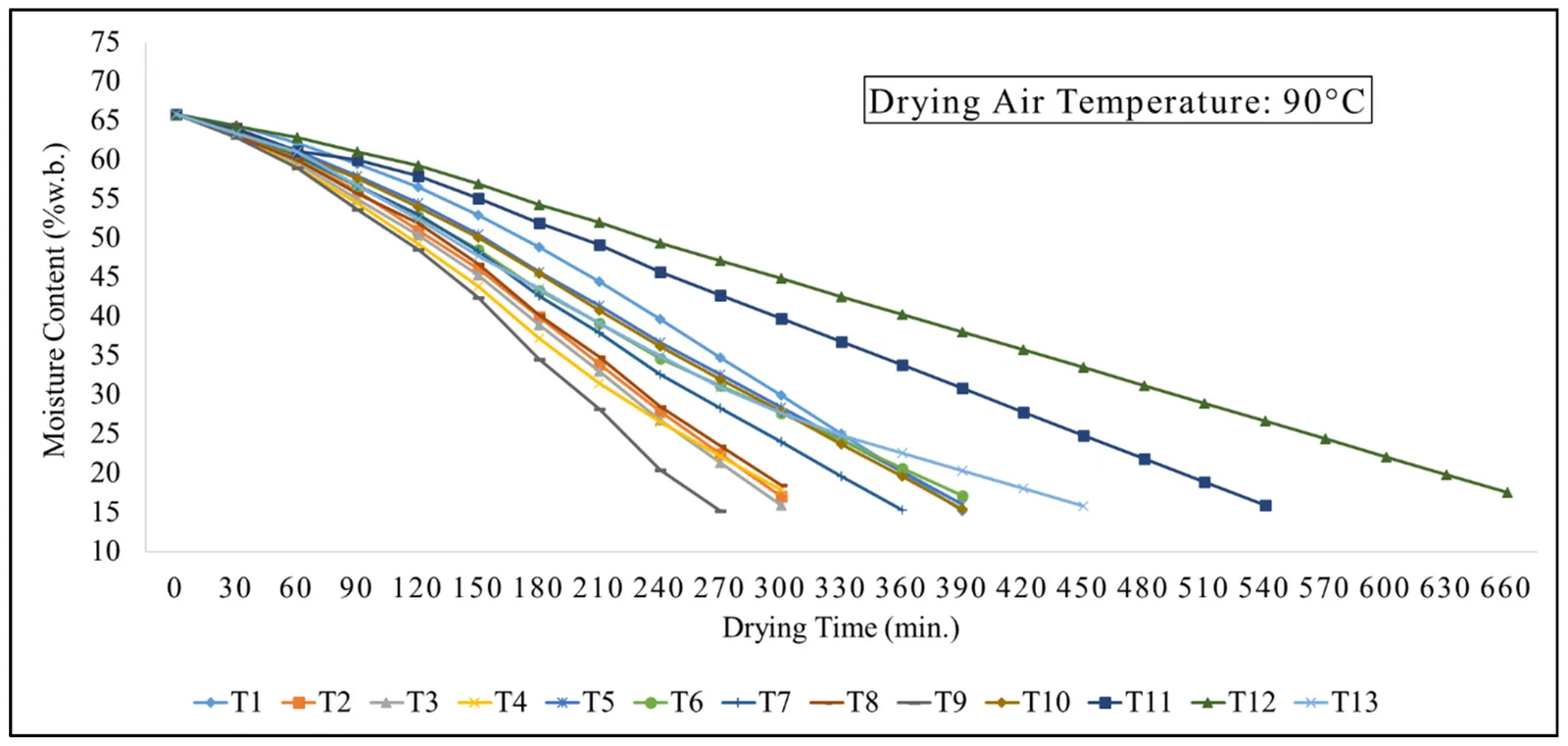
Descriptive drying curves of pretreated and untreated myrtle berries at 90 °C. Each curve represents one labeled tray containing an initial fruit load of 250 g, and mass measurements were recorded at 15 min intervals. All 13 treatment groups were dried simultaneously in the same temperature run. Tray positions were assigned and rearranged using a random-number table. The horizontal reference line indicates the target moisture content of 20% wet basis (wb). The curves represent single drying trays and do not constitute independent process replicates.

Increasing the drying temperature to 90 °C further shortened the overall drying process (Figure 4). The initial moisture content was approximately 65% wb. T9 (grape molasses + static immersion, 60 min) and the untreated control (T13) exhibited the fastest drying behavior, reaching approximately 15–20% moisture content after about 270–300 min. Most ultrasound-assisted treatments (T1–T6), together with T7 and T8, reached low moisture levels after approximately 300–390 min. T12 (water + static immersion, 60 min) again showed the slowest drying behavior and remained above the target moisture content of 20% wb at the end of the recorded drying period. Therefore, no extrapolated time to 20% wb was assigned to this treatment. T11 (water + static immersion, 45 min) also showed a slower drying tendency than most of the other treatments.

Overall, increasing the drying temperature from 60 to 90 °C shortened the observed drying period, while the ordering of the pretreatments varied with temperature. T1 and T2, both representing ultrasound treatment in grape molasses, showed the shortest observed drying times at 60 and 75 °C, respectively. At 90 °C, the shortest observed times were recorded for T9, representing static immersion in grape molasses, and the untreated control (T13). T12, representing static immersion in water for 60 min, showed the slowest observed drying behavior at all three temperatures. Thus, no single pretreatment produced the shortest drying time under all evaluated temperature conditions.

## 3. Discussion

The present study explored the drying behavior and measured quality attributes of myrtle berries subjected to ultrasound-assisted or static immersion in grape molasses or water, followed by forced-air convective drying at 60, 75, and 90 °C. Osmotic pretreatment is commonly used before drying because immersion in a hypertonic medium may promote partial water removal and simultaneous solute transfer [20,24,25], while ultrasound may further modify mass transfer through cavitation-related structural changes [26,27,28]. However, water loss and solid gain were not quantified in the present study. Moreover, each pretreatment–temperature combination was represented by one drying tray, and the triplicate compositional measurements were analytical rather than independent process replicates. The observed patterns are therefore interpreted descriptively. Overall, the measured quality profiles were condition- and compound-dependent, and no single pretreatment showed a consistent advantage across all drying temperatures and measured attributes.

### 3.1. Bioactive-Compound Patterns Among Pretreatment–Temperature Combinations

The untreated control showed the highest measured TAC concentration at all three drying temperatures. For many pretreatments, the concentrations measured at 60 °C were higher than those measured at 75 and 90 °C, which is broadly consistent with literature describing the thermal sensitivity of anthocyanins in blueberries, other berries, and myrtle fruits [29,30,31,32]. However, this pattern was not uniform across all treatments, and the present values describe analytical concentrations in single processed samples rather than statistically demonstrated treatment effects. Moreover, the results cannot be interpreted as true retention from fresh fruit because fresh-fruit TAC was expressed per unit volume of juice, whereas dried-sample TAC was expressed per unit dry weight.

The TPC response varied among the pretreatment–temperature combinations. T8 showed the highest measured TPC concentration at 60 °C, whereas the untreated control showed the highest concentrations at 75 and 90 °C. T6 and T12 showed comparatively high values at 90 °C, while T4 and T8 showed their highest measured values at 60 °C. Similar increases in extractable TPC under some intensive drying conditions have been reported previously [33,34,35,36]. Possible explanations discussed in the literature include increased extractability of bound phenolics following tissue disruption and differences in extraction efficiency [31,34,35,36,37]. Nevertheless, the present descriptive concentration data do not demonstrate improved phenolic preservation.

Several pretreated samples showed lower measured TPC concentrations than the untreated control, particularly at 75 and 90 °C. Leaching of water-soluble compounds during osmotic treatment has been reported previously [28,30]; however, water loss, solid gain, and phenolic transfer to or from the osmotic solution were not quantified in the present study. Therefore, this mechanism cannot be verified.

### 3.2. Sugar-Profile Patterns

The temperature-related glucose and fructose patterns differed among pretreatments. Several treatments showed lower measured glucose concentrations at 90 °C than at 60 or 75 °C, whereas fructose concentrations were frequently higher at 90 °C. However, these trends were not uniform across all pretreatments. Sucrose hydrolysis has been proposed as a possible explanation for changes in glucose and fructose during thermal processing [38]; nevertheless, the present concentration data do not independently demonstrate sugar conversion or degradation.

The treatment showing the highest measured total sugar concentration varied with drying temperature: T4 at 60 °C, T10 at 75 °C, and T2 at 90 °C. These values represent dry-weight-based concentrations rather than sugar enrichment relative to fresh fruit. Because fresh-fruit sugars were reported per unit volume of juice and the required juice-to-fruit conversion factors were unavailable, no fresh-to-dried concentration factor was calculated.

Ultrasound-assisted osmotic treatment can affect water and solute transfer through cavitation-induced structural changes [39,40,41,42,43,44,45], and grape molasses may provide an osmotic medium containing sugars, minerals, and phenolic compounds [46]. However, water loss, solid gain, and solute uptake were not quantified in the present study. These mechanisms are therefore presented as literature-based possibilities and not as experimentally verified explanations for the measured sugar or phenolic concentrations.

Overall, the treatment combinations produced different dried-product profiles. The untreated control showed the highest measured TAC concentration at all three temperatures, whereas the treatment showing the highest total sugar concentration differed with temperature: T4 at 60 °C, T10 at 75 °C, and T2 at 90 °C. No single treatment produced the highest measured values for all compositional attributes.

### 3.3. Drying-Behavior Patterns Under Different Pretreatments

The observed drying curves varied among the combinations of ultrasound application, immersion medium, treatment duration, and drying temperature. Ultrasound-assisted treatments generally showed shorter observed drying times than static-immersion treatments at 60 and 75 °C, but this pattern was not consistent at 90 °C. The T1–T3 groups were correctly defined as ultrasound treatments performed in grape molasses for 30, 45, and 60 min, respectively. Previous studies have associated ultrasound-assisted drying behavior with cavitation-induced microchannels and changes in tissue permeability [10,47]. However, tissue microstructure and effective moisture diffusivity were not measured in the present study; therefore, these mechanisms cannot be verified for the current treatments.

The treatment showing the shortest drying time varied with the drying temperature. At 60 °C, T1 (grape molasses + ultrasound, 30 min) exhibited the fastest drying behavior, whereas T2 (grape molasses + ultrasound, 45 min) showed the shortest drying time at 75 °C. At 90 °C, T9 (grape molasses + static immersion, 60 min) and the untreated control (T13) reached the target moisture range in the shortest time. In contrast, T12 (water + static immersion, 60 min) consistently exhibited the slowest drying behavior at all three temperatures. The rapid drying of T9 at 90 °C also indicates that the shortest observed drying time was not always associated with ultrasound treatment. Similarly, the slow drying behavior of T12 cannot be attributed to the formation of a sugar-rich surface layer because this treatment involved static immersion in water rather than grape molasses. Collectively, these observations suggest that drying behavior depended on the combined pretreatment conditions and drying temperature rather than on a single processing factor.

During osmotic treatment, water loss from fruit tissue may occur simultaneously with the uptake of soluble solids from the surrounding medium, while ultrasound may further modify both mass transfer processes [19,28]. However, water loss and solid gain were not quantified during the pretreatment stage in the present study. Therefore, mechanistic interpretations based on solute deposition, pore blockage, or crust formation cannot be verified for the treatments investigated. Moreover, although higher drying temperatures substantially shortened the drying time, processing efficiency should be evaluated together with potential changes in thermolabile bioactive compounds and other quality attributes [16,32,33]. Consequently, the selection of an appropriate pretreatment and drying temperature should be based on the intended product characteristics rather than drying time alone.

Overall, the findings of the present study should be interpreted in light of several limitations. Each pretreatment–temperature combination was represented by a single labeled drying tray in one drying run at each temperature; therefore, the observed drying-curve differences are descriptive, and possible run- and tray-related variability could not be evaluated statistically. Fresh-fruit TPC and Trolox-equivalent antioxidant activity were reported on a fresh-weight basis, whereas fresh-fruit TAC and sugars were reported per unit volume of juice. The corresponding dried-sample results were expressed on a dry-weight basis. A complete treatment-specific mass balance, including initial and final dry-matter amounts, water loss, solid gain, dry-matter recovery, juice yield, and juice density, was not established. Consequently, true compound retention, processing loss, enrichment, and solute uptake could not be calculated. The grape molasses was characterized by soluble solids, pH, and total acidity, but its detailed sugar, mineral, and phenolic composition was not determined. In addition, individual phenolic compounds were analyzed once per treatment–temperature combination, and volatile compounds were analyzed only in fresh fruit. The ultrasound treatments were conducted at 25 °C for a maximum of 60 min, limiting direct thermal exposure; nevertheless, the study did not include targeted analyses of oxidation products, process-induced contaminants, or potentially toxic compounds. Future studies should include independently replicated pretreatment and drying batches, complete mass transfer measurements, same-basis compositional analyses, replicated individual-phenolic measurements, detailed characterization of the osmotic medium, chemical-safety assessment, and sensory evaluation.

## 4. Materials and Methods

### 4.1. Plant Material and Experimental Design

Black myrtle (*M. communis* L. ‘Yakup’) fruits were harvested in November 2023 from three 13-year-old trees at the experimental orchard of Akdeniz University, Antalya, Türkiye (36°53′ N, 30°38′ E). For initial characterization, fruits from each tree were processed separately with three analytical determinations per tree. For the drying experiment, fruits collected from the three trees were pooled, mixed thoroughly, and allocated to 39 pretreatment–temperature combinations: 12 pretreatments (T1–T12) arranged in a 2 × 2 × 3 factorial structure [medium (grape molasses or water) × application technique (ultrasound or static immersion) × treatment duration (30, 45, or 60 min)] plus one untreated control (T13), each evaluated at 60, 75, and 90 °C. At each drying temperature, every treatment group was represented by one labeled drying tray. Consequently, each pretreatment–temperature combination corresponded to one processed and dried tray sample. Where triplicate compositional measurements were performed, these were analytical determinations obtained from the same dried tray sample rather than independent pretreatment or drying replicates.

### 4.2. Pretreatment Applications

The 12 active pretreatments were defined by a 2 × 2 × 3 factorial structure: Medium [commercial undiluted grape molasses (GM) vs. distilled water (W)] × Technique [ultrasound-assisted (US) vs. static immersion (N)] × Duration [30, 45, and 60 min]. This experimental structure was selected to permit descriptive comparison of the immersion medium, the presence or absence of ultrasound, and increasing treatment duration. Water-based treatments served as the comparison medium for the grape-molasses treatments, while static immersion treatments allowed the ultrasound-assisted treatments to be compared with immersion under otherwise similar conditions. The treatment durations of 30, 45, and 60 min were selected to represent progressively increasing exposure periods within the ranges commonly investigated for ultrasound-assisted osmotic pretreatment of fruit materials [18,20].

Grape molasses was selected as a natural, food-grade, high-soluble-solids medium with regional relevance and potential application in fruit processing [19,21]. Commercial undiluted grape molasses (Product code 07085046; İstanbul, Türkiye) was used. According to the product label, grape molasses was the sole ingredient. Before use, the molasses had a soluble solids content of 73.3 °Brix, a pH of 5.15, and a total titratable acidity of 0.41 g/100 g. Total acidity was determined according to Cemeroğlu [48]. The distilled water used in the water-based treatments had a measured pH of 5.8 ± 0.2.

The treatment codes were defined as follows: T1–T3, grape molasses + ultrasound for 30, 45, and 60 min, respectively; T4–T6, water + ultrasound for 30, 45, and 60 min, respectively; T7–T9, grape molasses + static immersion for 30, 45, and 60 min, respectively; and T10–T12, water + static immersion for 30, 45, and 60 min, respectively. T13 was the untreated control and received neither immersion nor ultrasound before drying.

For each active pretreatment, 1.0 kg of fruit was allocated to the corresponding treatment medium at a fruit-to-medium ratio of 1:5 (*w*/*v*). Ultrasound-assisted treatments were performed in an ultrasonic bath (ISOLAB Laborgeräte GmbH, Eschau, Germany; 40 kHz, 320 W) maintained at 25 °C, whereas static-immersion treatments followed the same immersion procedure without ultrasound activation. Following pretreatment, the fruits were drained at room temperature for approximately 2 min and gently blotted with filter paper to remove free surface liquid. The fruits were not rinsed. The untreated control was transferred directly to the drying stage without immersion or ultrasound.

### 4.3. Drying Procedure

Drying trials were conducted in a laboratory-scale forced-air convective tray dryer (Dalle LT-27 Digital, Dalle Engineering, Antalya, Türkiye; 1000 W; overall dimensions: 46.2 × 40.2 × 44.7 cm). The standard dryer contained 12 stainless-steel trays, and an additional labeled tray was fitted so that all 13 treatment groups could be dried simultaneously at each drying temperature. Each tray measured 39.0 × 28.3 cm. Following pretreatment, a 250 g fruit portion from each treatment was distributed in a single non-overlapping layer on one tray, corresponding to a loading density of approximately 2.3 kg/m^2^. Drying was conducted at 60, 75, or 90 ± 1 °C with a nominal air velocity of 1.5 m/s. The dryer was preheated until the target temperature reached steady-state conditions. Relative humidity was not measured. The labeled trays were assigned and rearranged among chamber positions using a random-number table to reduce systematic tray-position effects [37,49,50,51,52,53,54].

Sample mass was recorded at 15 min intervals using a GF-600 precision balance (A&D Company, Ltd., Tokyo, Japan) with 0.001 g readability. Trays were removed from the drying chamber for less than 2 min during each weighing and were immediately returned to the chamber. Initial moisture content was determined gravimetrically by oven drying a representative subsample at 70 °C to constant mass. Moisture content on a wet basis was calculated as MCwb (%) = [(mt − md)/mt] × 100, where mt is the sample mass at time t and md is the corresponding dry-matter mass. A moisture content of 20% wb was used as the reference drying endpoint based on Sumbul et al. [55]. When a treatment did not reach 20% wb during the recorded drying period, no endpoint time was extrapolated. Dried fruits were vacuum-sealed in polyethylene bags and stored at −80 °C for a maximum of four weeks before analysis.

### 4.4. Analytical Methods

Analytical standards and reagents used in the sugar, organic-acid, phenolic, anthocyanin, antioxidant, and volatile analyses were obtained from Sigma-Aldrich (St. Louis, MO, USA) or Merck (Darmstadt, Germany). Reagents were of analytical grade, and solvents used for chromatographic analyses were of HPLC grade. Ultrapure water was obtained using a Millipore purification system (Millipore Corp., Bedford, MA, USA).

#### 4.4.1. Pomological and Physicochemical Analyses

Fruit weight, width, and length were measured on 45 randomly selected fresh fruits in total, comprising 15 fruits from each of the three trees. Color parameters (L*, a*, b*) were determined using a chroma meter (CR-400, Konica Minolta, Inc., Tokyo, Japan).

In the CIELAB color system, L* represents lightness from black (0) to white (100), positive and negative a* values represent red and green, respectively, and positive and negative b* values represent yellow and blue, respectively [56]. Only the directly measured L*, a*, and b* coordinates were reported; chroma (C*) and hue angle (h°) were not calculated. Soluble solids content was measured using a digital refractometer (PAL-Bx, ATAGO Co., Ltd., Tokyo, Japan) and expressed as °Brix. Titratable acidity was determined according to Cemeroğlu [48] and expressed as g malic acid/100 g fresh fruit. pH was measured using an HI5221 digital pH meter (Hanna Instruments, Inc., Woonsocket, RI, USA).

#### 4.4.2. Bioactive Compounds and Antioxidant Activity

Total Phenolic Content (TPC): TPC was determined using the Folin–Ciocalteu method [57]. Fresh-fruit results were expressed as mg gallic acid equivalent (GAE)/100 g fresh weight (fw), whereas dried-sample results were expressed as mg GAE/100 g dry weight (dw).

Total Anthocyanin Content (TAC): TAC was measured using the pH-differential method [58]. Fresh-fruit results were expressed as mg cyanidin-3-glucoside equivalent/L juice, whereas dried-sample results were expressed as mg cyanidin-3-glucoside equivalent/100 g dw.

Antioxidant Activity (DPPH): DPPH radical-scavenging activity was determined according to Brand-Williams et al. [59]. The results were expressed both as percentage inhibition and as Trolox-equivalent antioxidant activity. Percentage inhibition represents the direct response obtained from the DPPH assay, whereas Trolox-equivalent values provide a standardized expression that facilitates comparison with studies reporting antioxidant capacity relative to a Trolox calibration. These two expressions were derived from the same analytical assay and were not treated as independent response variables. Fresh-fruit results were expressed as µmol TE/100 g fw, whereas dried-sample results were expressed as µmol TE/100 g dw.

Because the fresh and dried samples were reported on different analytical bases and treatment-specific dry-matter recovery was not included in a complete mass balance, the fresh and dried results were not used to calculate true retention percentages.

#### 4.4.3. Sugar and Organic Acid Profile by HPLC

Sugars (sucrose, glucose, and fructose) were analyzed by HPLC using a method modified from Miron and Schaffer [60]. Fresh-fruit juice samples were thawed at room temperature, and 1 mL of juice was mixed with 4 mL of ultrapure water. The mixtures were sonicated at 80 °C for 15 min, centrifuged at 5500 rpm for 15 min, and filtered through 0.45 µm, 13 mm nylon syringe filters before injection. Analyses were performed using a Prominence LC-20A system (Shimadzu Corporation, Kyoto, Japan) equipped with a refractive-index detector and a Coregel-87C column (7.8 × 300 mm; Concise Separations, San Jose, CA, USA). The column temperature was 70 °C, the mobile phase was ultrapure water under isocratic conditions, the flow rate was 0.6 mL/min, and the injection volume was 20 µL. External calibration was performed using Sigma-Aldrich sugar standards, and the available calibration record showed R^2^ = 0.9963. The sucrose standard and sucrose-spiked sample produced retention times of 8.787 and 8.791 min, respectively. Recovery was 92.19% for the 100 mg/L sucrose standard and 98.52% for the sucrose-spiked sample. Fresh-fruit results were expressed as mg/100 mL juice, whereas dried-sample results were expressed as mg/100 g dw.

Organic acids (citric, malic, and succinic acids) were determined only in fresh-fruit juice using a method modified from Bozan et al. [61]. One milliliter of juice was mixed with 4 mL of ultrapure water, shaken, centrifuged at 5500 rpm for 15 min, and filtered through 0.45 µm, 13 mm nylon syringe filters. Analyses were performed using a LC-20A HPLC system (Shimadzu Corporation, Kyoto, Japan) equipped with a UV detector and a Transgenomic 87H column (5 µm, 300 × 7.8 mm). The mobile phase was 0.01 N H_2_SO_4_ at a flow rate of 0.6 mL/min. The column temperature was 30 °C, the injection volume was 20 µL, and detection was performed at 210 nm. Organic acids were identified by comparing retention times and spectral data with Sigma-Aldrich standards and quantified using external calibration curves (R^2^ = 0.99978). Results were expressed as mg/100 mL fresh juice. Organic acids were not determined in dried samples and were not used to calculate processing retention or enrichment.

#### 4.4.4. Individual Phenolic Compounds by HPLC-DAD

Fourteen individual phenolic compounds were targeted: gallic, chlorogenic, caffeic, syringic, p-coumaric, ferulic, and ellagic acids; catechin; naringenin; myricetin; luteolin; kaempferol; cyanidin-3-glucoside; and pelargonidin-3-glucoside [62]. Nine compounds were quantified and included in Figure 1 and Supplementary Table S2: chlorogenic acid, caffeic acid, syringic acid, cyanidin-3-glucoside, pelargonidin-3-glucoside, ellagic acid, naringenin, myricetin, and catechin. Each pretreatment–temperature combination was analyzed once (n = 1). Source values recorded as zero represented non-detected compounds and are reported as n.d. in Supplementary Table S2.

For exploratory visualization, the full-precision concentration data for the nine individual phenolic compounds presented in Figure 1 were normalized separately across the 39 pretreatment–temperature combinations using min–max scaling. The normalized value for each observation was calculated as x_normalized_ = (x − x_min_)/(x_max_ − x_min_), where x_min_ and x_max_ represent the minimum and maximum measured concentrations of the corresponding compound, respectively. Thus, normalized values of 0 and 1 represented the minimum and maximum measured concentrations within each compound. For normalization only, observations reported as n.d. were assigned a numerical value of zero.

Agglomerative hierarchical clustering of both the individual phenolic compounds and the pretreatment–temperature combinations was performed on the normalized data using Ward’s linkage method and Euclidean distance. The heatmap was generated solely for exploratory visualization. The corresponding unscaled concentration values, expressed as mg/100 g dw and reported to two decimal places, are provided in Supplementary Table S2.

#### 4.4.5. Volatile Compounds by HS-SPME/GC-MS

Volatile compounds were analyzed only in fresh-fruit samples by HS-SPME/GC-MS using a method modified from Kraujalyte et al. [63]. One gram of fruit homogenate was placed in a headspace vial, and 1 mL of CaCl_2_ solution was added. Samples were equilibrated at 40 °C for 15 min, followed by extraction for 30 min at the same temperature using a 75 µm CAR/PDMS fiber. The fiber was desorbed in the GC injector at 260 °C for 5 min. Analyses were performed using a GC-2010 Plus GC-MS system (Shimadzu Corporation, Kyoto, Japan) equipped with an HP-Innowax column (Agilent Technologies, Santa Clara, CA, USA) (30 m × 0.25 mm internal diameter, 0.25 µm film thickness). Helium was used as the carrier gas at 1.0 mL/min, with a split ratio of 1:10. The oven temperature was initially maintained at 40 °C, increased to 260 °C at 5 °C/min, and held at 260 °C for 40 min. Mass spectra were acquired under electron ionization at 70 eV over m/z 30–400. Compounds were tentatively identified by comparison with Wiley and NIST mass-spectral libraries and expressed as relative peak-area percentages. Retention indices, authentic-standard confirmation, and a numerical library-match threshold were not available; therefore, the identifications are reported as tentative. Dried samples were not analyzed by HS-SPME/GC-MS, and no inference regarding aroma retention or processing-induced volatile changes was made. Min–max normalization, hierarchical clustering, and heatmap visualization were performed using Python version 3.12.13 (Python Software Foundation, Beaverton, OR, USA), with NumPy version 2.3.5, pandas version 2.2.3, SciPy version 1.17.0, seaborn version 0.13.2, and Matplotlib version 3.10.8.

### 4.5. Statistical Analysis

Fresh-fruit pomological and color characteristics are presented as mean ± SD based on 45 fruits in total (15 fruits from each of three trees). Fresh-fruit chemical measurements were based on three analytical determinations from each tree (n = 9), except for volatile compounds, for which one analysis was performed per tree (n = 3). DPPH antioxidant activity, TPC, TAC, and sugar composition of the dried samples were measured in triplicate from the same dried tray sample. These triplicate measurements represent analytical repeatability and not independent pretreatment or drying replication. Individual phenolic compounds were analyzed once per pretreatment–temperature combination (n = 1).

Descriptive statistics, including means and standard deviations, were calculated using IBM SPSS Statistics for Windows, version 23.0 (IBM Corp., Armonk, NY, USA). Each pretreatment–temperature combination in the drying experiment was represented by one labeled drying tray. Repeated mass measurements recorded at 15 min intervals were observations from the same tray and were not independent replicates. Consequently, no inferential statistical comparisons among pretreatments or drying temperatures are reported. ANOVA and Tukey’s HSD results, *p*-values, significance letters, and effect-size estimates are therefore not presented. Results are reported descriptively as measured values or as mean ± SD of analytical determinations. The individual phenolic-compound dataset and clustered heatmap were treated solely as exploratory and hypothesis-generating.

## 5. Conclusions

The measured quality attributes and drying behavior of myrtle berries varied among the pretreatment–temperature combinations. Ultrasound treatment in grape molasses for 30 and 45 min showed the shortest observed drying times at 60 and 75 °C, respectively. These observations identify T1 and T2 as candidate conditions for future independently replicated trials when shortening the drying process is the principal processing objective. However, this advantage was temperature-dependent, because static immersion in grape molasses for 60 min and the untreated control showed the shortest drying times at 90 °C.

No pretreatment consistently maximized all measured compositional attributes. The untreated control showed the highest measured TAC concentration at all three temperatures and the highest TPC concentration at 75 and 90 °C. Therefore, the pretreatments did not provide a uniform advantage in terms of phenolic, anthocyanin, antioxidant, or sugar concentrations. The principal potential benefit indicated by the present data is a condition-specific reduction in drying time rather than general preservation or enrichment of the measured compounds. Pretreatment and drying conditions should consequently be selected according to the intended product characteristic.

Because each pretreatment–temperature combination was represented by one processed and dried sample, and the triplicate determinations were analytical rather than independent process replicates, these findings are descriptive. Furthermore, water loss, solid gain, true compound retention, processing loss, enrichment, and solute uptake could not be quantified because a complete process mass balance and same-basis fresh-to-dried compositional measurements were unavailable. Future studies should include independently replicated pretreatment and drying batches, complete mass transfer measurements, detailed characterization of the osmotic medium, same-basis compositional analyses, replicated individual-phenolic measurements, chemical-safety assessment, and sensory evaluation.

## Figures and Tables

**Table 1:** Pomological, physicochemical, and bioactive characteristics of fresh ‘Yakup’ myrtle (*Myrtus communis* L.) fruits.

Parameter	Mean ± SD	Range (Min–Max)
Pomological		
Fruit Weight (g)	0.47 ± 0.07	0.32–0.61
Fruit Width (mm)	8.64 ± 0.77	6.82–10.08
Fruit Length (mm)	9.02 ± 0.75	7.15–10.81
Color		
L* (lightness)	30.23 ± 1.53	27.07–33.91
a* (red-green)	6.30 ± 1.02	3.48–7.93
b* (yellow-blue)	7.85 ± 1.35	3.92–9.79
Physicochemical		
SSC (°Brix)	17.82 ± 2.75	15.00–22.30
Titratable acidity (g malic acid/100 g fw)	0.97 ± 0.32	0.53–1.31
pH	4.23 ± 0.27	3.92–4.57
Bioactive Compounds		
Total Phenolics (mg GAE/100 g fw)	605.99 ± 173.73	377.24–758.93
Total Anthocyanins (mg/L cy-3-glu)	94.56 ± 18.07	70.69–113.44
DPPH (% Inhibition)	86.63 ± 0.56	85.59–87.32
DPPH (μmol TE/100 g fw)	77.03 ± 0.56	75.99–77.72

SSC: Soluble Solids Content; fw: fresh weight; cy-3-glu: cyanidin-3-glucoside equivalent. Pomological/color: n = 45 (3 trees × 15 fruits); Chemical analyses: n = 9 (3 trees × 3 replicates).

**Table 2:** Sugar, organic acid, and volatile compound profile of fresh ‘Yakup’ myrtle fruits.

Parameter	Mean ± SD	Range (Min–Max)
Sugars (mg/100 mL juice)		
Glucose	5153.47 ± 512.94	4600.16–5799.12
Fructose	5702.28 ± 546.12	5187.15–6416.07
Sucrose	n.d.	–
Total Sugars	10,855.75 ± 1055.54	9823.52–12,213.44
Organic Acids (mg/100 mL juice)		
Citric Acid	674.82 ± 109.05	567.86–817.30
Malic Acid	744.14 ± 144.20	595.92–933.75
Succinic Acid	2631.96 ± 272.16	2294.01–2973.52
Total Organic Acids	4050.92 ± 317.08	3784.17–4509.63
* Volatile Compounds (% peak area)		
Alcohols	59.82 ± 1.53	58.17–61.04
Eucalyptol	41.14 ± 0.19	40.94–41.32
α-Terpineol	5.55 ± 0.47	5.17–6.11
Terpenes	19.59 ± 2.58	16.77–21.82
α-Pinene	12.75 ± 0.66	12.21–13.51
Esters	13.12 ± 1.44	11.48–14.35
(-)-8-p-Menthen-2-yl acetate	5.85 ± 1.15	4.48–6.77
Linalyl acetate	4.42 ± 1.58	2.74–5.90
Aldehydes	3.54 ± 1.11	2.25–4.26
Ketones	0.92 ± 0.17	0.78–1.12
Other Compounds	3.01 ± 2.26	1.27–5.67
Total Identified	100.00	100.00

* Sugar and organic-acid data are expressed as mean ± SD of nine analytical determinations (three trees × three analytical determinations). Volatile compounds were analyzed once per tree (n = 3) by HS-SPME/GC-MS and are reported as relative peak-area percentages. Volatile identifications are tentative. n.d.: not detected.

## Data Availability

The original contributions presented in this study are included in the article/Supplementary Material. Further inquiries can be directed to the corresponding author.

## Supplementary Materials

The following supporting information can be downloaded at: https://www.mdpi.com/article/10.3390/plants15172616/s1, Table S1. Complete volatile-compound profile of fresh ‘Yakup’ myrtle (*Myrtus communis* L.) fruits (relative peak-area percentages). Table S2. Individual phenolic-compound concentrations (mg/100 g dw) in dried ‘Yakup’ myrtle (*Myrtus communis* L.) fruits under different pretreatments (T1–T13) and drying temperatures (60, 75, and 90 °C). Each pretreatment–temperature combination was analyzed once (n = 1); the data are exploratory.

## Abbreviations

CAR/PDMS	carboxen/polydimethylsiloxane
DAD	diode-array detector
DPPH	2,2-diphenyl-1-picrylhydrazyl
dw	dry weight
fw	fresh weight
GAE	gallic acid equivalents
GC–MS	gas chromatography–mass spectrometry
GM	grape molasses
HPLC	high-performance liquid chromatography
HS-SPME	headspace solid-phase microextraction
N	static immersion without ultrasound
n.d.	not detected
OD	osmotic dehydration
RID	refractive-index detector
SD	standard deviation
SSC	soluble solids content
TAC	total anthocyanin content
TE	Trolox equivalents
TPC	total phenolic content
UAOP	ultrasound-assisted osmotic pretreatment
US	ultrasound-assisted treatment
W	water
wb	wet basis
ACN	acetonitrile
MCwb	moisture content on a wet basis
